# The Quaternization Reaction of 5-*O*-Sulfonates of Methyl 2,3-*o*-Isopropylidene-*β*-D-Ribofuranoside with Selected Heterocyclic and Aliphatic Amines

**DOI:** 10.3390/molecules25092161

**Published:** 2020-05-05

**Authors:** Barbara Dmochowska, Rafał Ślusarz, Jarosław Chojnacki, Justyna Samaszko-Fiertek, Janusz Madaj

**Affiliations:** 1 Carbohydrate Chemistry Group, Faculty of Chemistry, University of Gdańsk, 80-308 Gdańsk, Poland; rafal.slusarz@ug.edu.pl (R.Ś.); j.samaszko-fiertek@ug.edu.pl (J.S.-F.); janusz.madaj@ug.edu.pl (J.M.); 2 Department of Inorganic Chemistry, Technical University of Gdańsk, 80-233 Gdańsk, Poland; jaroslaw.chojnacki@pg.edu.pl

**Keywords:** sugar sulfonates, quaternary ammonium salt, methyl 2,3-*O*-isopropylidene-D-ribofuranoside, heterocyclic amines, X-ray crystallography

## Abstract

The synthesis of *N*-((methyl 5-deoxy-2,3-*O*-isopropylidene-*β*-D-ribofuranoside)-5-yl)ammonium salts are presented. To determine the effect of the nucleophile type and outgoing group on the quaternization reaction, selected aliphatic and heterocyclic aromatic amines reacted with: methyl 2,3-*O*-isopropylidene-5-*O*-tosyl-*β*-D-ribofuranoside or methyl 2,3-*O*-isopropylidene-5-*O*-mesyl-*β*-D-ribofuranoside or methyl 2,3-*O*-isopropylidene-5-*O*-triflyl-*β*-D-ribofuranoside were performed on a micro scale. High-resolution ^1^H- and ^13^C-NMR spectral data for all new compounds were recorded. Additionally, the single-crystal X-ray diffraction analysis for methyl 2,3-*O*-isopropylidene-5-*O*-mesyl-*β*-D-ribofuranoside and selected in silico interaction models are reported.

## 1. Introduction

**Figure 1:**
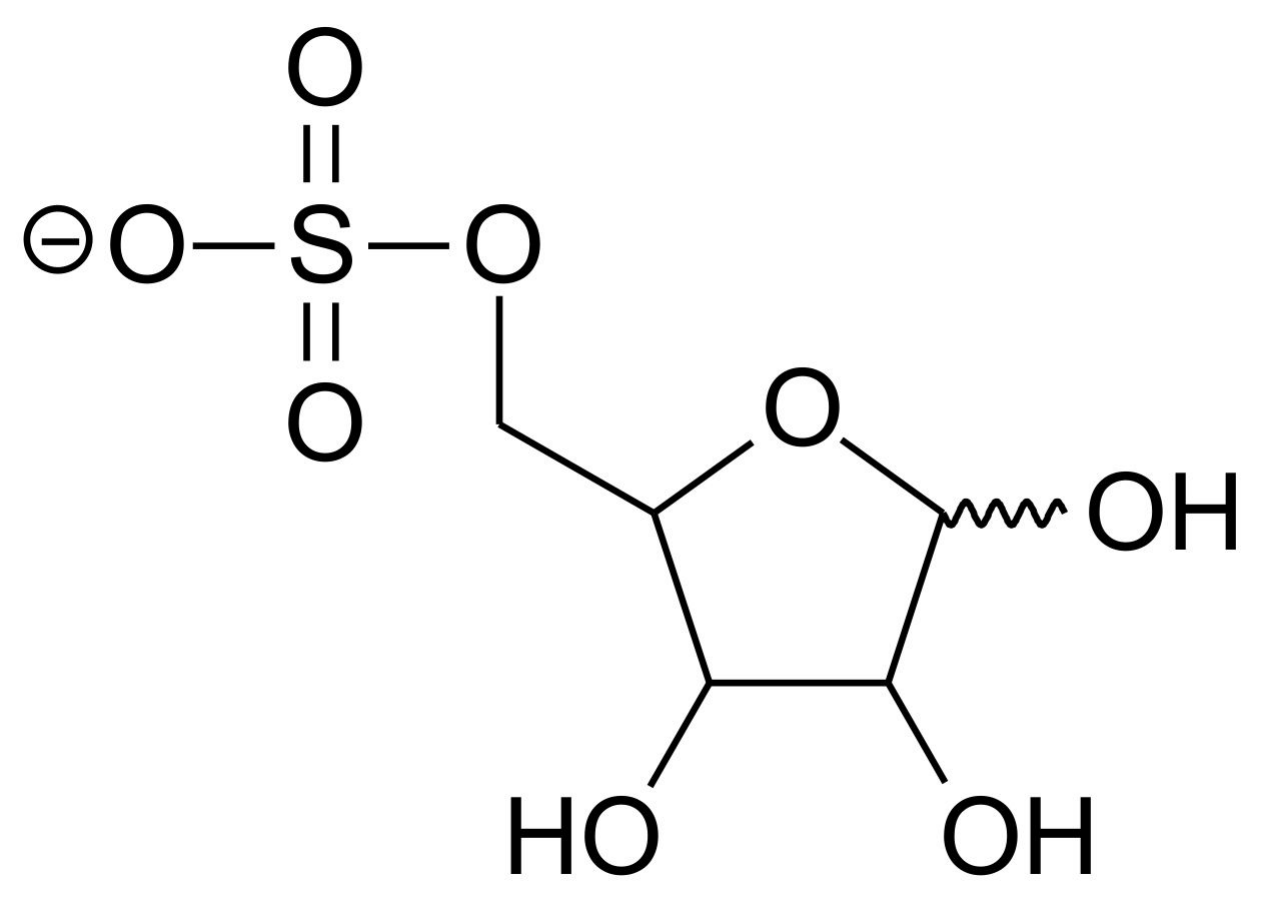
Sulfate ester analog of D-ribose phosphate monoester.

Preparation of sulfate esters is one of the very useful reactions in organic chemistry. It was first described by Adolph Strecker in 1868 [1]. Additionally, in the synthesis of carbohydrates, the synthesis of sulfonates has been widely described. In 1953, Tipson described exhaustively the preparation of such derivatives using sulfonyl halides in pyridine [2]. This method is one of the most commonly used methods until now. Another described in the literature method of obtaining esters of *p*-toluenesulfonic acids is based on the use of an anhydride of this acid [3,4]. Another remarkable method is the use of silver methanesulfonate as the sulfonating agent [5,6]. The interest in sulfonating sugars results from the biological activity of this type of derivatives. Among the glycosaminoglycans with high medicine and industry usage potential, there should be mentioned heparan sulfate, keratan sulfate, dermatan sulfate and chondroitin sulfate. The last one [7] is widely used in the treatment of osteoarthritis resulting in improvements in physical function, quality of life and reductions in pain and disease progression. Another naturally occurring sulfated polysaccharide is heparin. It was discovered accidentally in 1959 by McLean [8] and was initially used clinically as a drug in the field of treatment thrombosis and hemostasis in the 1930s and 1940s. Since it has been shown that the low-molecular heparin is highly active, attempts have been made to develop methods for its synthesis [9,10]. The presence of large amounts of simple sugars in the diet of a modern man is supposed to result in a possible disruption of their metabolic pathways. For example, disturbances in the hexosamine biosynthetic pathway may be associated with type 2 diabetes, Alzheimer’s and cancer [11,12]. Hyperactivity in the pentose pathway and glycolysis may result in cancer [13,14,15,16]. An important role in regulating this type of abnormality is associated with naturally occurring derivatives of monosaccharides such as glucose-6-sulphate [17]. For some time, researchers have been interested in sulfate analogues of ribose phosphate (see Figure 1) [18,19,20,21].

Its simple synthesis from methyl 5-deoxy-5-iodo-2,3-*O*-isopropylidene-D-riboside was described by Musicki and Widlanski [22]. Use of very active methyl 5-deoxy-5-*O*-trifluoromethanesulfonate-2,3-*O*-isopropylidene-D-riboside led to low yield and a complicated mixture of products.

We describe the synthesis of similar sulphate derivatives, which we then quaternized to obtain the appropriate quaternary ammonium salts (QAS) with different counterion. Studies show that different counterions can result in different biological activities of ammonium salts [23,24].

## 2. Results and Discussion

This work is an extension of our previously published results of calculations regarding the conformations and mechanisms of formation of quaternary ammonium salts with the results of experimental work. Both types of data show very high convergence, which further increases their value [25,26].

### 2.1. Synthesis of 4b–4k, 6a–6f and 6i and 8b–8d and 8f

**Scheme 1:**
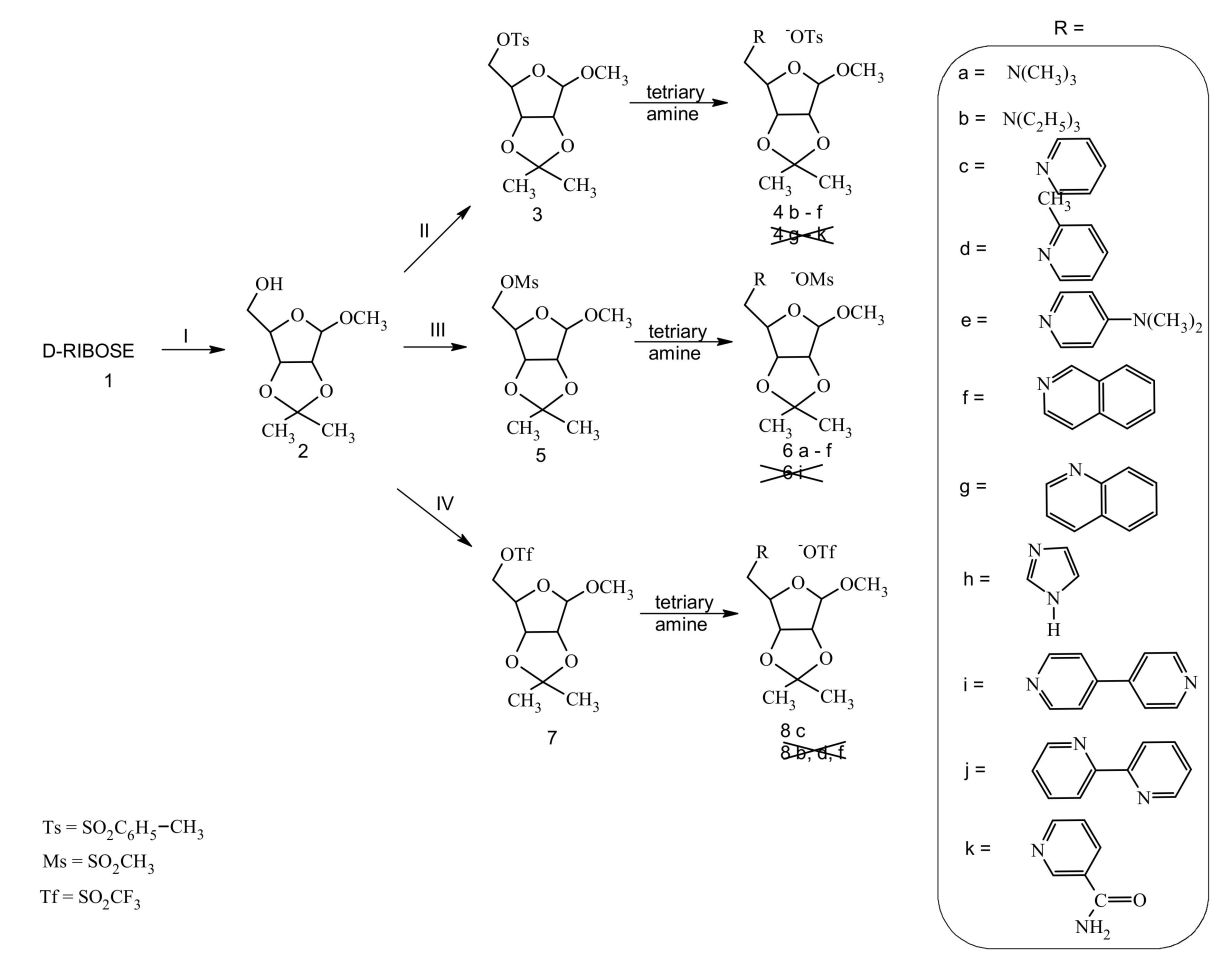
Formation of *N*-((methyl 5-deoxy-2,3-*O*-isopropylidene-*β*-D-ribofuranoside)-5-yl)ammonium sulfonates. Reagents: (**I**) acetone, MeOH, SnCl_2_·2H_2_O, conc. H_2_SO_4_; (**II**) Py, TsCl, 0–5 °C, 16 h; (**III**) CH_2_Cl_2_, Py, MsCl, 0–5 °C, 16 h; (**IV**) CH_2_Cl_2_, Tf_2_O, −50 °C, 15 min.

The idea was to carry out the reaction methyl 2,3-*O*-isopropylidene-5-*O*-tosyl-*β*-D-ribofuranoside (**3**) or methyl 2,3-*O*-isopropylidene-5-*O*-mesyl-*β*-D-ribofuranoside (**5**) or methyl 2,3-*O*-isopropylidene-5-*O*-triflyl-*β*-D-ribofuranoside (**7**) with tertiary amines such as triethylamine, trimethylamine, 4-(*N*,*N*-dimethylamine)pyridine, quinoline, isoquinoline, 2-methylpyridine, pyridine, imidazole, 4,4-bipyridyl, 2,2-bipyridyl and 3-carbamoylpyridine (numbering according to Scheme 1, below). We were able to obtain quaternary ammonium salts at the primary atom in the furanose ring.

To confirm our assumptions, we used a series of tertiary amines for reactions, ranging from aliphatic to heterocyclic with two rings.

Based on our greatest experience from the reactions of forming quaternary ammonium salts, we started to work with methyl 2,3-*O*-isopropylidene-5-*O*-tosyl-*β*-D-ribofuranoside (**3**). All newly synthesized *N*-((methyl 5-deoxy-2,3-*O*-isopropylidene-*β*-D-ribofuranoside)-5-yl)ammonium salts were found to be water-soluble. Their structures have been fully confirmed using NMR spectroscopy.

Methyl 2,3-*O*-isopropylidene-5-*O*-tosyl-*β*-D-ribofuranoside (**3**) [27] was obtained as a white solid in 92% yield. Its reaction yielding to *N*-((methyl 5-deoxy-2,3-*O*-isopropylidene-*β*-D-ribofuranoside)-5-yl)-*N*,*N*,*N*-trimethylammonium tosylate (**4a**) and crystallographic structure has already been described by us [28].

In this work, we have expanded the research of compound **3** to include reactions with other amines. Reaction of this compound with triethylamine in CH_3_CN yielded *N*-((methyl 5-deoxy-2,3-*O*-isopropylidene-*β*-D-ribofuranoside)-5-yl)-*N*,*N*,*N*-triethylammonium tosylate (**4b**) as an oil (with 28% yield only). This reaction was carried out for 14 days at 70 °C.

The reaction of methyl 2,3-*O*-isopropylidene-5-*O*-tosyl-*β*-D-ribofuranoside (**3**) with aromatic amines pyridine, 2-methylpyridine, 4-(*N*,*N*-dimethylamine)pyridine or isoquinoline led to the formation of corresponding *N*-((methyl 5-deoxy-2,3-*O*-isopropylidene-*β*-D-ribofuranoside)-5-yl)ammonium salts (**4c**–**4f**). The respective yields of these reactions are shown in Table 1.

Reaction of compound **3** with pyridine led to *N*-((methyl 5-deoxy-2,3-*O*-isopropylidene-*β*-D-ribofuranoside)-5-yl)pyridinium tosylate (**4c**). After 14 days of the reaction at 70 °C, the expected product was obtained with a yield of 78%. An analogous reaction with 2-methylpyridine allowed to receive **4d** only with a yield of 31%. This result clearly indicates the effect of steric hindrance of the methyl substituent at the C-2 position of the amine on the substitution of the sulfonic group on the terminal group of the sugar derivative **3**. After the reaction was completed, the substrate **3** was present in the reaction mixture, next to the expected product **4d**, and the prolongation of the reaction time did not improve its yield.

Since 4-(*N*,*N*-dimethylamine)pyridine (DMAP) is a solid, the reaction with **3** was initially carried out in acetonitrile solution at 70 °C for a month, which lead to the desired salt with surprisingly low yield. After the chromatographic purification of the post-reaction mixture, the expected salt was obtained in a yield of 37%. In addition, we found the unreacted substrate in the mixture. Since DMAP is both a stronger base and a nucleophile than pyridine, which is the result of the presence of the dimethylammonium residue in the *para* position, we expected a greater or at least similar quaternization efficiency as for pyridine.

The obtained efficiency came to us as a surprise and prompted us to attempt a synthesis as in the case of pyridine and 2-methylpyridine, so without the use of a solvent. In the next synthesis, we decided to use the melting method developed and published by us for other quaternary ammonium salts. Usually, it leads to the expected products with good yields. The substrate **3** and DMAP were heated in a screw cap ampoule at 100 °C for 48 h. This time, after purification, we obtained the product *N*-((methyl 2,3-*O*-isopropylidene-*β*-D-ribofuranoside)-5-yl)-4-(*N*,*N*-dimethylamine)pyridine tosylate (**4e**) as the pale yellow oil with a yield of 70%. We did not find any substrate in the post-reaction mixture. The result obtained by this method coincided with our expectations and matched logically to a number of previously obtained results.

In addition to heterocyclic monocyclic amines, we used two heterocyclic bicyclic amines in subsequent syntheses. The quaternization reaction using isoquinoline required a much longer time (216 days compared to 14) but allowed us to obtain *N*-((methyl 5-deoxy-2,3-*O*-isopropylidene-*β*-D-ribofuranoside)-5-yl)isoquinolinium tosylate (**4f**) in a similar 72% yield as for pyridinium salt (**4c**). This result could be explained by the similar basicity (pK_a_ 5.23 of pyridine and pK_a_ 5.42 of isoquinoline [29]) and similar nucleophilicity of both amines.

Under analogous conditions, the reaction of the substrate tosylate 3 with quinoline led only to trace amounts of the expected product *N*-((methyl 5-deoxy-2,3-*O*-isopropylidene-*β*-D-ribofuranoside)-5-yl)quinolinium tosylate (**4g**). This result can be explained by the less basicity of quinoline (pK_a_ 4.90 [29]) and greater steric hindrance of the nitrogen atom.

We also carried out the reactions of the tosylate **3** with the following amines: imidazole, 4,4-bipyridyl, 2,2-bipyridyl and 3-carbamoylpyridine. Reactions were carried out in both acetonitrile solutions at 70 °C and solvent-free at 100 °C, and in all cases, we observed a lack of products and only unreacted tosylate **3**.

Summing up the reaction of the methyl 2,3-*O*-isopropylidene-5-*O*-tosyl-*β*-D-ribofuranoside (**3**) with selected amines, we can say that we were able to obtain and characterize six new quaternary ammonium salts. Yields for obtaining *N*-((methyl 2,3-*O*-isopropylidene-*β*-D-ribofuranoside)-5-yl)ammonium tosylates ranged from 28% to 78%. The highest yield was obtained for the synthesis of *N*-((methyl 2,3-*O*-isopropylidene-*β*-D-ribofuranoside)-5-yl)pyridine tosylate (**4c**). It came as no surprise to us that the lowest performance was obtained in the case of *N*-((methyl 2,3-*O*-isopropylidene-*β*-D-ribofuranoside)-5-yl)triethylammonium tosylate (**4b**) (Table 1) [30]. Such results are expected to be caused by the large steric hindrance of the nitrogen atom in the amine molecule and, thus, the difficult substitution of the terminal *O*-tosyl group.

To compare the effect of the leaving group on the quaternization reactions, we conducted reactions of selected amines with the mesyl analogue of compound **3**. Another reason was to study the effect of counterion on the biological properties of the salts obtained.

Therefore, 2,3-*O*-isopropylidene-*β*-D-ribofuranoside (**2**) was treated with mesyl chloride in pyridine at 0 °C and *N*-((2,3-*O*-isopropylidene-*β*-D-ribofuranoside methyl)-5-yl)ammonium mesylates (**5**) was obtained with 57% yield as the pale yellow crystals. Due to the case of the tosyl derivative **3,** only six amines allowed to obtain a positive result, we also decided to use them, i.e., trimethylamine, triethylamine, pyridine, 2-methylpyridine, 4-(*N*,*N*-dimethylamino)pyridine and isoquinoline, in reactions with mesyl derivative **5**.

Reaction of 2,3-*O*-isopropylidene-5-*O*-mesyl-*β*-D-ribofuranoside (**5**) with ethanolic trimethylamine solution gave the desired salt in good 72% yield, which was very similar to that obtained in the case of tosylate **3** (see Table 1).

Methyl 2,3-*O*-isopropylidene-5-*O*-mesyl-*β*-D-ribofuranoside (**5**) was reacted with trimethylamine in an ethanolic solution. The yield of this synthesis (compound **6a**) was 72% and was close to the analogous reaction of 5-*O*-tosylderivative (**4a**) with trimethylamine (see Table 1).

Compared to the reaction of compound **3**, in the case of mesylate **5**, we extended the reaction time with triethylamine to 30 days, also leading it at 70 °C, and yet, we obtained the product **6b** in only 19% yield. The lower yield despite the longer reaction time was undoubtedly the result of steric hindrance caused by three ethyl groups attached to the amine nitrogen atom.

The reaction of compound **5** with pyridine was carried out analogously to that of compound **3** –14 days at 70 °C, leading to *N*-((2,3-*O*-isopropylidene-*β*-D-ribofuranoside methyl)-5-yl)pyridinium mesylate (**6c**) obtained in 66% yield. In this case, the yield was noticeably lower.

A similarly lower result was obtained as a result of the quaternization of compound **5** using 2-methylpyridine. In this case, we obtained *N*-((2,3-*O*-isopropylidene-*β*-D-ribofuranoside methyl)-5-yl)-2-methylpyridinium mesylate (**6d**) with 19% yield (31% for compound **3**). Further prolongation of the reaction time did not bring any improvement in the reaction efficiency.

Due to the case of the reaction of tosylate **3** with DMAP, we obtained much better reactions results using a procedure reaction in a closed ampoule without solvent; a similar procedure was used for compound **5**. After 30 days of reaction, we were able to get the *N*-((2,3-*O*-isopropylidene-*β*-D-ribofuranoside methyl)-5-yl)-4-(*N*,*N*-dimethylamine)pyridinium mesylate (**6e**) with 55% yield, and further extension of the retraction time did not improve it.

Heating of compound **5** in the presence of isoquinoline for 14 days at 70 °C led to the formation of *N*-((2,3-*O*-isopropylidene-*β*-D-ribofuranoside methyl)-5-yl)isoquinoline mesylate (**6f**) in slightly lower yield than in the case of tosylate **3** (**4f** compared to 72%).

Very high reactivity as a leaving group of *O*-trifluoromethanesulfone is widely known. We have synthesized 2,3-*O*-isopropylidene-5-*O*-triflyl-*β*-D-ribofuranoside (**7**) by acting on methyl 2,3-*O*-isopropylidene-*β*-D-ribofuranoside (**2**) with trifluoromethanesulfonic anhydride, which, due to high reactivity and low durability, was reacted without isolation with isoquinoline, 2-methylpyridine, pyridine and triethylamine (Scheme 1). Despite many attempts of purification, we managed to obtain and characterize only *N*-((2,3-*O*-isopropylidene-*β*-D-ribofuranoside methyl)-5-yl)pyridinium triflate (**8c**) with a very high yield of 98%.

In the quaternization reactions we carried out with selected amines, the triflyl derivative proved to be not very useful. It could possibly be the result of the side reactions that this very reactive derivative underwent.

### 2.2. Molecular Modeling

**Figure 2:**
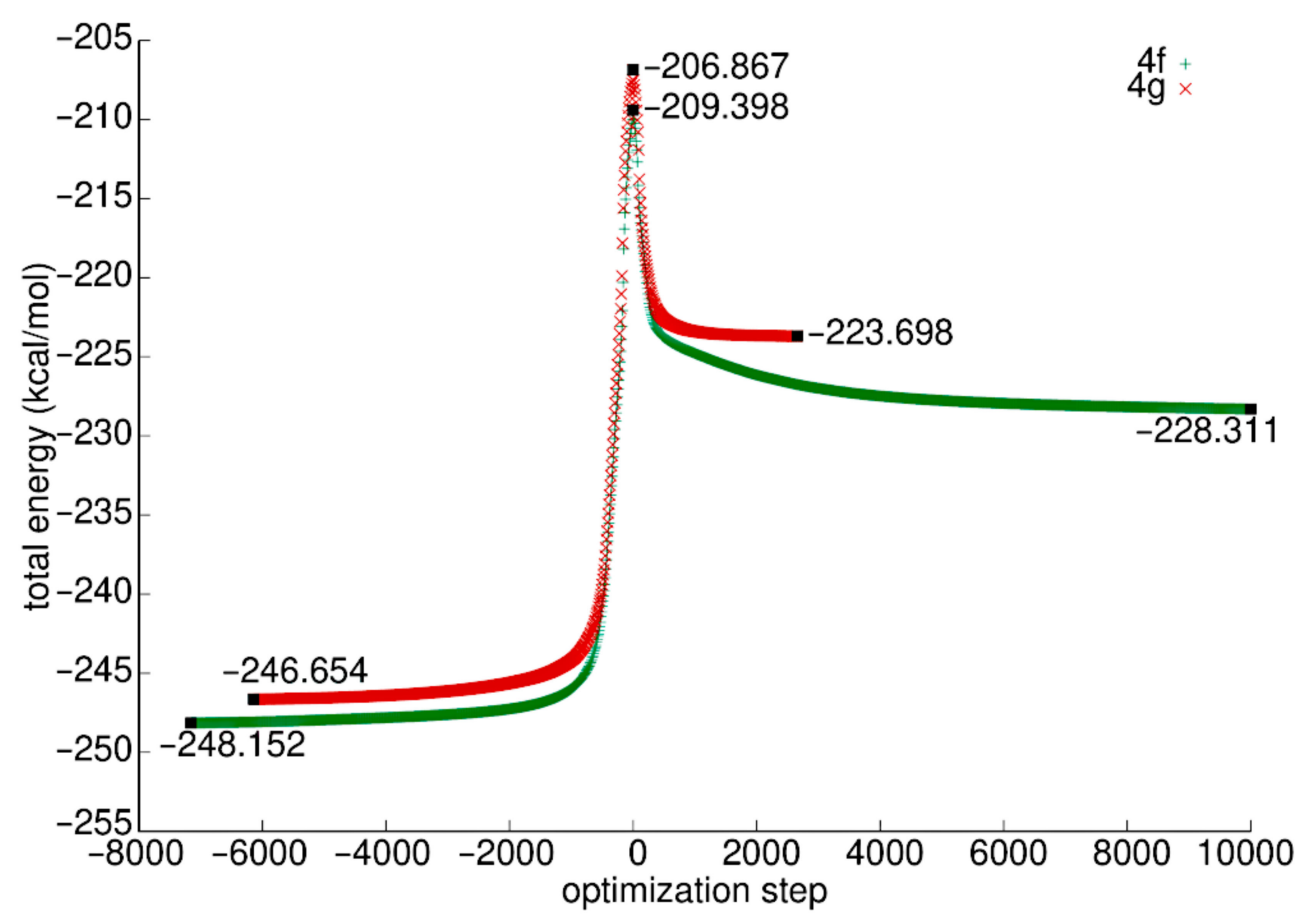
Energetics of synthesis of **4f** (green pluses) and **4g** (red x’s). Total energies of substrates are on the left side of the plot (negative count of optimization steps), and total energies of products are on the right side of the plot. Total energies of both transition state complexes are in step 0.

The difference in the reactivity of isoquinoline and quinoline was explained with semi-empirical calculation using the PM6 Hamiltonian [31] in mopac2016 [32]. The tosylate **3** with quinoline were geometry optimized and subjected to the reaction path calculations in mopac2016. After finding, confirming and optimizing the transitional state (TS) complexes, we have calculated the reaction path in two directions: from TS towards the products (**4f** and tosyl or **4g** and tosyl, respectively) and from TS towards the substrates (tosylate **3** and isoquinoline or tosylate **3** and quinoline, respectively). In both cases, the total energy of simulated complexes was measured and visualized in Figure 2.

The resulting energies of both substrates and products of synthesis of **4g** are higher than corresponding energies calculated for **4f** (~1.5 kcal/mol and ~4.6 kcal/mol for substrates and products, respectively). Moreover, the energetic barrier between substrates and TS complexes is over 1 kcal/mol higher in the case of **4g** than in the case of **4f**. Energy gained after the transition from TS into products side is almost 5 kcal/mol higher for the **4f** set of products comparing to the **4g** mixture. All 3D models (Protein Data Bank format) of **4f** and **4g** discussed here (TS, products and substrates of both) are available in the Supplementary Materials.

### 2.3. X-ray Diffraction Analysis of 5

**Figure 3:**
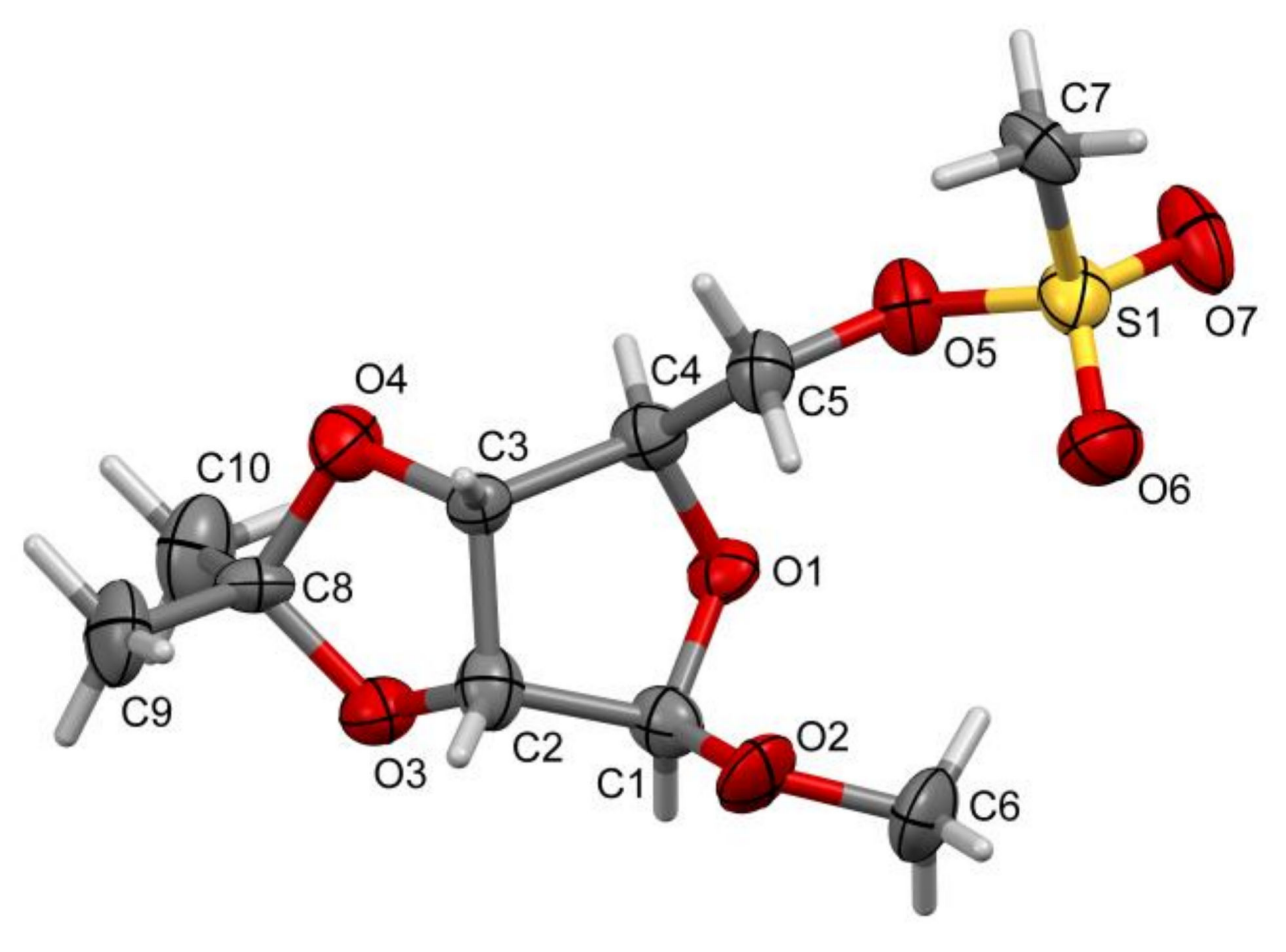
Molecular view and atom-labeling scheme for the X-ray structure of methyl 2,3-*O*-isopropylidene-5-*O*-mesyl-*β*-D-ribofuranoside. Displacement ellipsoids drawn at the 50% probability level.

The crystallographic structure of methyl 2,3-*O*-isopropylidene-5-*O*-mesyl-*β*-D-ribofuranoside (**5**), which was a substrate for the preparation of quaternary ammonium salts, was determined and is shown in Figure 3.

With high probability, it can be assumed, which is confirmed by the aforementioned calculation results, that this structure corresponds to that in the solution. Since the quaternization reaction follows the S_N_2 mechanism, it could be expected that the steric factor would have a significant impact on the course of the reaction next to the electronic factors.

## 3. Materials and Methods

Commercial D-ribose (Merck, New Jersey, NJ, USA) was used. All reactions were monitored by thin-layer chromatography (TLC) on Kieselgel 60 F254 Silica Gel plates (E. Merck, 0.20 mm thickness) using eluent system (v/v) 3:1 CHCl3-MeOH (Merck). The spots were detected by spraying with 5% ethanolic H2SO4 and charring. Other reagents, such as C2H5OH, H2SO4, 2-butanon, tertiary amines, CH3CN, CH2Cl2, Tf2O were obtained from Merck. 1H-NMR and 13C-NMR spectra were recorded at 25 °C with a Varian Mercury (Agilent, Santa Clara, CA, USA) spectrometer at 400.49 and 100.70 MHz, respectively, with Me4Si as the internal standard; positive-ion mode MALDITOF mass spectra was done on a Bruker Biflex III spectrometer (Billerica, Massachusetts, MA, USA).

### 3.1. General Procedure for Synthesis of Quaternary Ammonium Salts

Methyl 2,3-*O*-isopropylidene-5-*O*-tosyl-*β*-D-ribofuranoside (**3**) or methyl 2,3-*O*-isopropylidene-5-*O*-mesyl-*β*-D-ribofuranoside (**5**) or methyl 2,3-*O*-isopropylidene-5-*O*-triflyl-*β*-D-ribofuranoside (**7**) was dissolved in a tertiary amine or in a solution of amine. The mixture was conditioned in a screw capped ampoule at 70 °C (or 100 °C), after which, it was evaporated to dryness. The residue was dissolved in H_2_O and extracted with CHCl_3_. The aqueous layer was concentrated on the reduced pressure and crystallized from 2-butanone. *R*_f_ = 0 (3:1 CHCl_3_-MeOH).

### 3.2. Procedure for 4a–4f

*N-((methyl 5-deoxy-2,3-O-isopropylidene-β-D-ribofuranoside)-5-yl)trimethylammonium tosylate* (**4a**). Compound **3** (150 mg, 0.42 mmol) was dissolved in 33% ethanolic solution of trimethylamine (0.31 mL) and conditioned for 48h. After crystallization from 2-butanon, **4a** was obtained (117.5 mg, 67%); mp. 75–76 °C; (α)*_D_*^20^ −12.00 (*c* 0.2, H_2_O); ^1^H-NMR (D_2_O): δ 7.71 and 7.39 (2d, each 2H, Ph), 5.22 (s, 1H, H-1), 4.89 (dd, 1H, H-3, *J*_2,3_ 5.6; *J*_3.4_ 1.4), 4.81 (d, 1H, H-2, *J*_2,3_ 6.0), 4.77 (m, 1H, H-4, *J*_4,5_′ 9.6), 3.69 (dd, 1H, H-5, *J*_4,5_ 2.6, *J*_5,5_′ 14.0), 3.55 (dd, 1H, H-5′, *J*_4,5_′ 9.6), 3.49 (s, 3H, -OCH_3_), 3.26 (s, 9H, N(CH_3_)_3_), 2.42 (s, 3H, PhCH_3_), 1.56 and 1.40 (2s, each 3H, C(CH_3_)_2_); ^13^C-NMR (H_2_O) δ 129.66 and 125.70 (C, Ph), 114.08 (C, *C*(CH_3_)_2_), 110.42 (C-1), 83.86 (C-2), 83.08 (C-3), 81.16 (C-4), 68.91 (C-5), 56.32 (C, O*C*H_3_), 54.26 (C, N(CH_3_)_3_), 25.62 and 24.02 (C, C(*C*H_3_)_2_), 20.70 (C, Ph*C*H_3_); MALDI TOF- MS (CCA): *m*/*z* 246 ((M-OTs)^+^).

*N-((methyl 5-deoxy-2,3-O-isopropylidene-β-D-ribofuranoside)-5-yl)triethylammonium tosylate* (**4b**). Compound **3** (10 mg, 0.03 mmol) was dissolved in dry triethylamine (0.02 mL). After 14 days, compound **4b** was obtained as an oil (35.7 mg, 28%); (α)*_D_*^20^ 8.00 (*c* 0.2, H_2_O); ^1^H-NMR (D_2_O): δ 7.71 and 7.38 (2d, each 2H, Ph), 5.18 (s, 1H, H-1), 4.85 (dd, 1H, H-3, *J*_2,3_ 6.0; *J*_3.4_ 2.2), 4.78 (d, 1H, H-2, *J*_2,3_ 5.6), 4.61 (m, 1H, H-4), 3.48 (m, 2H, H-5 and H-5′), 3.48 (s, 3H, -OCH_3_), 3.40 and 1.30 (15H, NEt_3_), 2.41 (s, 3H, PhCH_3_), 1.55 and 1.39 (2s, each 3H, C(CH_3_)_2_); ^13^C-NMR (H_2_O) δ129.68 and 125.65 (C, Ph), 114.33 (C, *C*(CH_3_)_2_), 110.30 (C-1), 83.87 (C-2), 83.25 (C-3), 81.41 (C-4), 59.90 (C-5), 56.61 (C, O*C*H_3_), 53.98-46.93 (C, N(CH_2_CH_3_)_3_), 25.73 and 24.14 (C, C(*C*H_3_)_2_), 20.71 (C, Ph*C*H_3_); MALDI TOF- MS (CCA): *m*/*z* 288.3 ((M-OTs)^+^).

*N-((methyl 5-deoxy-2,3-O-isopropylidene-β-D-ribofuranoside)-5-yl)pyridinium tosylate* (**4c**). Compound **3** (30 mg, 0.084 mmol) was dissolved in dry pyridine (0.02 mL). After 14 days, compound **4c** was obtained as an oil (28.5 mg; 78%); (α)*_D_*^20^ 24.00 (*c* 0.2, H_2_O); ^1^H-NMR (D_2_O): δ 8.87; 8.66 and 8.13 (5H, Ph), 7.69 and 7.37 (2d, each 2H, Ph), 5.17 (s, 1H, H-1), 5.04 (d, 1H, H-3, *J*_2,3_ 5.8), 4.98 (dd, 1H, H-5, *J*_5,5_′ 14), 4.92 (d, 1H, H-2, *J*_2,3_ 5.8), 4.79 (dd, 1H, H-4, *J*_4,5_ 3.6; *J*_4,5_′ 10.8), 4.69 (dd, 1H, H-5′, *J*_4,5_′ 10.8), 3.46 (s, 3H, -OCH_3_), 2.41 (s, 3H, PhCH_3_), 1.52 and 1.41 (2s, each 3H, C(CH_3_)_2_); ^13^C-NMR (H_2_O) δ 146.76; 139.77; 128.67 (Ph-amine), 129.66 and 125.61 (C, Ph), 113.98 (C, *C*(CH_3_)_2_), 110.75 (C-1), 85.08 (C-4), 84.32 (C-2), 81.41 (C-3), 63.52 (C-5), 56.43 (C, O*C*H_3_), 25.52 and 24.02 (C, C(*C*H_3_)_2_), 20.70 (C, Ph*C*H_3_); MALDI TOF- MS (CCA): *m*/*z* 266.1 ((M-OTs)^+^).

*N-((methyl 5-deoxy-2,3-O-isopropylidene-β-D-ribofuranoside)-5-yl)-2-methylpyridinium tosylate* (**4d**). Compound **3** (0.30 mg; 0.084 mmol) was added to 2-methylpyridine (0.18 mL). After 14 days, compound **4d** was obtained as an oil (11.16 mg, 31%): (α)*_D_*^20^ 32.50 (*c* 0.2, H_2_O); ^1^H-NMR (D_2_O): δ 8.72 and 7.93 (4H, Ph), 7.70 and 7.38 (2d, each 2H, Ph), 5.20 (s, 1H, H-1), 5.06 (dd, 1H, H-3, *J*_3,4_ 1.2), 4.99 (dd, 1H, H-5, *J*_4,5_ 3.8; *J*_5,5_′ 14), 4.94 (d, 1H, H-2, *J*_2,3_ 6.0), 4.76 (m, 1H, H-4), 4.65 (dd, 1H, H-5′, *J*_4,5_′ 3.8), 3.41 (s, 3H, -OCH_3_), 2.92 (s, 3H, PhCH_3_-amine), 2.42 (s, 3H, PhCH_3_), 1.53 and 1.42 (2s, each 3H, C(CH_3_)_2_); ^13^C-NMR (H_2_O) δ 146.19–142.55; 130.71 and 125.71 (Ph-amine), 129.63 and 125.60 (C, Ph), 114.06 (C, *C*(CH_3_)_2_), 110.78 (C-1), 83.68 (C-4), 84.32 (C-2), 81.66 (C-3), 59.80 (C-5), 56.49 (C, O*C*H_3_), 25.58 and 24.06 (C, C(*C*H_3_)_2_), 20.70 (C, Ph*C*H_3_); MALDI TOF- MS (CCA): *m*/*z* 280.2 ((M-OTs)^+^).

*N-((methyl 5-deoxy-2,3-O-isopropylidene-β-D-ribofuranoside)-5-yl)-4-(N,N-dimethylamino)-pyridinium tosylate (**4e**)*.

Procedure 1. Compound **3** (30 mg; 0.084 mmol) and 4-(*N*,*N*-dimethylamino)pyridine (5.2 mg) was melted and conditioned at 100 °C for 48 h. Compound **4e** was obtained as a light oil (14.5 mg; 70%).

Procedure 2. Compound **3** (36 mg; 0.1 mmol) and 4-(*N*,*N*-dimethylamino)pyridine (6.15 mg) were dissolved in CH_3_CN (1.5 mL). The solution was conditioned at 70°C for a month. Compound **4e** was obtained as an oil (9.1 mg; 37%); (α)*_D_*^20^ 21.50 (*c* 0.2, H_2_O); ^1^H-NMR (D_2_O): δ 7.95 and 6.87 (4H, Ph), 7.67 and 7.33 (2d, each 2H, Ph), 5.14 (s, 1H, H-1), 4.93 (dd, 1H, H-3, *J*_3,4_ 0.8), 4.87 (d, 1H, H-2, *J*_2,3_ 5.6), 4.63 (dd, 1H, H-4, *J*_4,5_ 4.6), 4.40 (dd, 1H, H-5, *J*_5,5_′ 14.8), 4.12 (m, 1H, H-5′), 3.44 (s, 3H, -OCH_3_), 3.19 (s, 6H, N(CH_3_)_2_), 2.38 (s, 3H, PhCH_3_), 1.52 and 1.40 (2s, each 3H, C(CH_3_)_2_); ^13^C-NMR (H_2_O) δ 141.66 and 107.89; (Ph-amine), 129.61 and 125.58 (C, Ph), 113.83 (C, *C*(CH_3_)_2_), 110.47 (C-1), 85.10 (C-4), 84.48 (C-2), 81.35 (C-3), 59.44 (C-5), 56.30 (C, O*C*H_3_), 39,64 (C, N(*C*H_3_)_2_), 25.54 and 24.00 (C, C(*C*H_3_)_2_), 20.70 (C, Ph*C*H_3_); MALDI TOF- MS (CCA): *m*/*z* 309.4 ((M-OTs)^+^).

*N-((methyl 5-deoxy-2,3-O-isopropylidene-β-D-ribofuranoside)-5-yl)isoquinolinium tosylate* (**4f**). Compound **3** (30 mg; 0.084 mmol) was added to isoquinoline (0.23 g). After 9 days, compound **4f** was obtained as a light oil (29.9 mg, 72%); (α)*_D_*^20^ 23.00 (*c* 0.2, H_2_O); ^1^H-NMR (D_2_O): δ 9.65–8.04 (7H, Ph), 7.63 and 7.28 (2d, each 2H, Ph), 5.16 (s, 1H, H-1), 5.08 (m, 2H, H-3, H-5), 4.93 (d, 1H, H-2, *J*_2,3_ 5.6), 4.89 (dd, 1H, H-4, *J*_3,4_ 3.6, *J*_4,5_ 10.4), 4.77 (dd, 1H, H-5′, *J*_5,5_′ 13.4), 3.45 (s, 3H, -OCH_3_), 2.35 (s, 3H, PhCH_3_), 1.52 and 1.42 (2s, each 3H, C(CH_3_)_2_); ^13^C-NMR (H_2_O) δ 149.96–130.43 and 127.78–126.93 (Ph-amine), 129.57–125.53 (C, Ph), 113.98 (C, *C*(CH_3_)_2_), 110.79 (C-1), 85.03 (C-4), 84.43 (C-2), 81.50 (C-3), 63.18 (C-5), 56.47 (C, O*C*H_3_), 25.52 and 24.01 (C, C(*C*H_3_)_2_), 20.65 (C, Ph*C*H_3_); MALDI TOF- MS (CCA): *m*/*z* 316.1 ((M-OTs)^+^).

### 3.3. Procedure for 6a–6f

*N-((methyl 5-deoxy-2,3-O-isopropylidene-β-D-ribofuranoside)-5-yl)trimethylammonium mesylate* (**6a**). Compound **5** (44 mg, 0.14 mmol) was dissolved in a 33% ethanolic solution of trimethylamine (0.12 mL) and conditioned for 120h. After crystallization from 2-butanon, **6a** was obtained (38.6 mg, 72%); mp. 153-155°C; (α)*_D_*^20^ -24.00 (*c* 0.2, H_2_O); ^1^H-NMR (D_2_O): δ 5.24 (s, 1H, H-1), 4.91 (dd, 1H, H-3, *J*_3.4_ 1.6), 4.83 (d, 1H, H-2, *J*_2,3_ 6.0), 4.79 (m, 1H, H-4), 3.71 (dd, 1H, H-5, *J*_4,5_ 2.4, *J*_5,5_′ 14.0), 3.57 (dd, 1H, H-5′, *J*_4,5_′ 9.6), 3.49 (s, 3H, -OCH_3_), 3.28 (s, 9H, N(CH_3_)_3_), 2.83 (s, 3H, -CH_3_), 1.57 and 1.41 (2s, each 3H, C(CH_3_)_2_); ^13^C-NMR (H_2_O) δ 114.08 (C, *C*(CH_3_)_2_), 110.44 (C-1), 83.88 (C-2), 83.10 (C-3), 81.17 (C-4), 68.92 (C-5), 56.34 (C, O*C*H_3_), 54.29 (C, N(CH_3_)_3_), 38.72 (*C*H_3_-), 25.65 and 24.06 (C, C(*C*H_3_)_2_); MALDI TOF- MS (CCA): *m*/*z* 246.2 ((M-OMs)^+^).

*N-((methyl 5-deoxy-2,3-O-isopropylidene-β-D-ribofuranoside)-5-yl)triethylammonium mesylate* (**6b**). Compound **5** (40 mg, 0.14 mmol) was dissolved in dry triethylamine (0.12 mL). After 30 days, compound **6b** was obtained as an oil (10.8 mg, 19%); (α)*_D_*^20^ -0.8 (*c* 0.2, H_2_O); ^1^H-NMR (D_2_O): δ 5.21 (s, 1H, H-1), 4.90 (dd, 1H, H-3, *J*_2,3_ 6.0; *J*_3.4_ 2.0), 4.81 (d, 1H, H-2, *J*_2,3_ 5.6), 4.61 (m, 1H, H-4), 3.48 (m, 2H, H-5 and H-5′), 3.48 (s, 3H, -OCH_3_), 3.48-3.20 and 1.30 (15H, NEt_3_), 2.83 (s, 3H, -CH_3_), 1.55 and 1.40 (2s, each 3H, C(CH_3_)_2_); MALDI TOF- MS (CCA): *m*/*z* 288.2 ((M-OMs)^+^).

*N-((methyl 5-deoxy-2,3-O-isopropylidene-β-D-ribofuranoside)-5-yl)pyridinium mesylate* (**6c**). Compound **5** (40 mg, 0.04 mmol) was dissolved in dry pyridine (0.26 mL).After 14 days, compound **6c** was obtained as an oil (33.8 mg; 66%); (α)*_D_*^20^ 16.40 (*c* 0.2, H_2_O); ^1^H-NMR (D_2_O): δ 8.91–8.16 (t,t,d, 5H, Ph), 5.19 (s, 1H, H-1), 5.07 (dd, 1H, H-3, *J*_3,4_ 1.2), 5.01 (dd, 1H, H-5, *J*_5,5_′ 13.2), 4.94 (d, 1H, H-2, *J*_2,3_ 6.0), 4.76 (m, 1H, H-4, *J*_4,5_ 5.0; *J*_4,5_′ 10.4), 4.67 (m, 1H, H-5′), 3.46 (s, 3H, -OCH_3_), 2.83 (s, 3H, -CH_3_), 1.53 and 1.42 (2s, each 3H, C(CH_3_)_2_); ^13^C-NMR (H_2_O) δ 146.68–128.70 (Ph-amine), 113.99 (C, *C*(CH_3_)_2_), 110.75 (C-1), 85.10 (C-4), 84.33 (C-2), 81.41 (C-3), 63.53 (C-5), 56.43 (C, O*C*H_3_), 38.67 (*C*H_3_-), 25.51 and 24.01 (C, C(*C*H_3_)_2_); MALDI TOF- MS (CCA): *m*/*z* 266.2 ((M-OMs)^+^).

*N-((methyl 5-deoxy-2,3-O-isopropylidene-β-D-ribofuranoside)-5-yl)-2-methylpyridinium mesylate* (**6d**). Compound **5** (44 mg; 0.14 mmol) was dissolved in 2-methylpyridine (0.3 mL). After 14 days, compound **6d** was obtained as an oil (11.30 mg, 19%): (α)*_D_*^20^ 10.90 (*c* 0.2, H_2_O); ^1^H-NMR (D_2_O): δ 8.75 and 7.94 (4H, Ph), 5.21 (s, 1H, H-1), 5.07 (dd, 1H, H-3, *J*_3,4_ 1.0), 4.99 (dd, 1H, H-5, *J*_4,5_ 3.8;), 4.95 (d, 1H, H-2, *J*_2,3_ 6.0), 4.76 (m, 1H, H-4), 4.66 (dd, 1H, H-5′, *J*_5,5_′ 11.2), 3.41 (s, 3H, -OCH_3_), 2.93 (s, 3H, PhCH_3_-amine), 2.84 (s, 3H, -CH_3_), 1.53 and 1.42 (2s, each 3H, C(CH_3_)_2_); ^13^C-NMR (H_2_O) δ 146.24–125.76 (Ph-amine), 114.07 (C, *C*(CH_3_)_2_), 110.78 (C-1), 84.34 (C-2), 83.71 (C-4), 81.67 (C-3), 59.81 (C-5), 56.48 (C, O*C*H_3_), 38.68 (*C*H_3_-), 25.57 and 24.05 (C, C(*C*H_3_)_2_), 20.06 (C, Ph*C*H_3_); MALDI TOF- MS (CCA): *m*/*z* 280.3 ((M-OMs)^+^).

*N-((methyl 5-deoxy-2,3-O-isopropylidene-β-D-ribofuranoside)-5-yl)-4-(N,N-dimethylamino)-pyridinium mesylate* (**6e**). Compound **5** (42 mg; 0.15 mmol) was added to 4-(*N*,*N*-dimethylamino)pyridine (9.15 mg) and conditioned. After 30 days, compound **6e** was obtained as an oil (33.3 mg; 55%); (α)*_D_*^20^ 16.5 (*c* 0.2, H_2_O); ^1^H-NMR (D_2_O): δ 8.05 and 6.96 (4H, Ph), 5.15 (s, 1H, H-1), 4.97 (dd, 1H, H-3, *J*_3,4_ 1.0), 4.89 (d, 1H, H-2, *J*_2,3_ 6.0), 4.67 (dd, 1H, H-4, *J*_4,5_ 4.8, *J*_4,5_′ 10.4), 4.46 (dd, 1H, H-5, *J*_5,5_′ 14.0), 4.16 (m, 1H, H-5′), 3.44 (s, 3H, -OCH_3_), 3.25 (s, 6H, N(CH_3_)_2_), 2.82 (s, 3H, -CH_3_), 1.53 and 1.41 (2s, each 3H, C(CH_3_)_2_); ^13^C-NMR (H_2_O) δ 141.75 and 107.93; (Ph-amine), 113.83 (C, *C*(CH_3_)_2_), 110.49 (C-1), 85.11 (C-4), 84.50 (C-2), 81.36 (C-3), 59.47 (C-5), 56.31 (C, O*C*H_3_), 39,70 (C, N(*C*H_3_)_2_), 38.71 (*C*H_3_-), 25.56 and 24.02 (C, C(*C*H_3_)_2_); MALDI TOF- MS (CCA): *m*/*z* 309.2 ((M-OMs)^+^).

*N-((methyl 5-deoxy-2,3-O-isopropylidene-β-D-ribofuranoside)-5-yl)isoquinolinium mesylate* (**6f**). Compound **5** (42 mg; 0.15 mmol) was added to isoquinoline (0.44 g). After 14 days, compound **6f** was obtained as a light oil (39.1 mg, 63%); (α)*_D_*^20^ 20.20 (*c* 0.2, H_2_O); ^1^H-NMR (D_2_O): δ 9.68–8.03 (7H, Ph), 5.16 (s, 1H, H-1), 5.11 (m, 2H, H-3, H-5, *J*_4,5_ 4.0), 4.94 (d, 1H, H-2, *J*_2,3_ 6.0), 4.90 (dd, 1H, H-4, *J*_4,5_′ 10.8), 4.77 (m, 1H, H-5′, *J*_5,5_′ 13.8), 3.45 (s, 3H, -OCH_3_), 2.81 (s, 3H, -CH_3_), 1.52 and 1.42 (2s, each 3H, C(CH_3_)_2_); ^13^C-NMR (H_2_O) δ 150.00–126.93 (Ph-amine), 113.96 (C, *C*(CH_3_)_2_), 110.82 (C-1), 85.07 (C-4), 84.43 (C-2), 81.51 (C-3), 63.22 (C-5), 56.48 (C, O*C*H_3_), 38.71 (*C*H_3_-), 25.55 and 24.03 (C, C(*C*H_3_)_2_); MALDI TOF-MS (CCA): *m*/*z* 316.2 ((M-OMs)^+^).

### 3.4. Procedure for 8c

*N-((methyl 5-deoxy-2,3-O-isopropylidene-β-D-ribofuranoside)-5-yl)pyridinium triflate* (**8c**). To a solution of compound **2** (100 mg; 0.49 mmol) in dichloromethane (5.5 mL), pyridine (0.7 mL) was added. The mixture was cooled to -50°C and then triflic anhydride (0.17 mL) in dry dichloromethane (3.2 mL) were added dropwise. After 15 min, the reaction was complete, and compound **8c** was isolated as an oil (200 mg; 98%); (α)*_D_*^20^ 11.00 (*c* 0.2, H_2_O); ^1^H-NMR (D_2_O): δ 8.91–8.17 (5H, Ph), 5.19 (s, 1H, H-1), 5.07 (d, 1H, H-3, *J*_2,3_ 6.4), 5.01 (dd, 1H, H-5, *J*_5,5_′ 13.6), 4.94 (d, 1H, H-2, *J*_2,3_ 6.4), 4.82 (m, 1H, H-4, *J*_4,5_ 4.0; *J*_4,5_′ 10.8), 4.67 (m, 1H, H-5′), 3.48 (s, 3H, -OCH_3_), 1.53 and 1.42 (2s, each 3H, C(CH_3_)_2_); ^13^C-NMR (H_2_O) δ 146.81-128.70 (Ph-amine), 113.99 (C, *C*(CH_3_)_2_), 110.76 (C-1), 85.10 (C-4), 84.33 (C-2), 81.41 (C-3), 63.54 (C-5), 56.43 (C, O*C*H_3_), 25.52 and 24.02 (C, C(*C*H_3_)_2_), 38.67 (*C*H_3_-), MALDI TOF-MS (CCA): *m*/*z* 266.3 ((M-OTf)^+^).

## 4. Conclusions

New quaternary ammonium salts, derivatives of methyl *β*-D-ribofuranoside (**2**), were obtained. There were no significant differences in the yields of the reactions for the preparation of the corresponding tosylates and mesylates. Pyridinium and trimethylammonium salts were easier prepared than salts of more spatially developed amines. Amines with an electron-donating substituent in the aromatic ring were more easily quaternized (e.g., DMAP) than amines with an electron-withdrawing substituent (e.g., 3-carbamoylpyridine). The steric factor proved to be extremely important in our quaternization reactions. This is significantly affected by the direction of the reaction in which the nucleophile, which is the tertiary amine, attacks from the opposite side to the sulfone group. Since it is outside the sugar ring, the nucleophile attack is hindered and occurs from above the furan ring, which makes the more difficult the attack the larger the nucleophile is. It seems that the results of the synthesis of 1,4-anhydo-2,3-*O*-isopropylidene-5-*O*-tosyl-D,L-ribitol (previously published [31]) contained in Table 2 provide good confirmation of this thesis. The lack of the *O*-methyl substituent at the sugar ring reduces the steric hindrance and facilitates the attack from the sugar ring side, resulting in better quaternization efficiency with the same amines.

## Figures and Tables

**Table 1:** The quaternization reaction yields.

Sulfate Ester	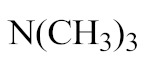	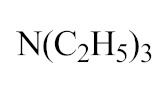	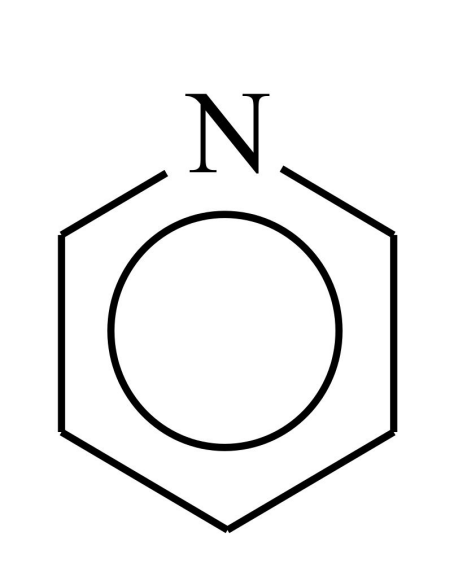	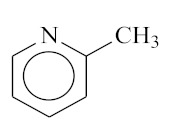	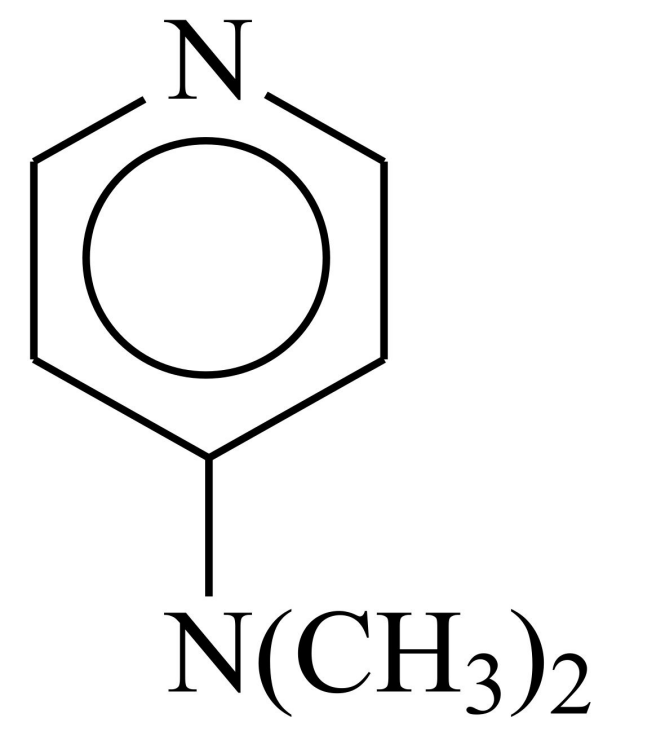	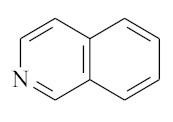	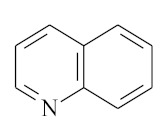	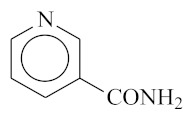	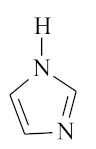
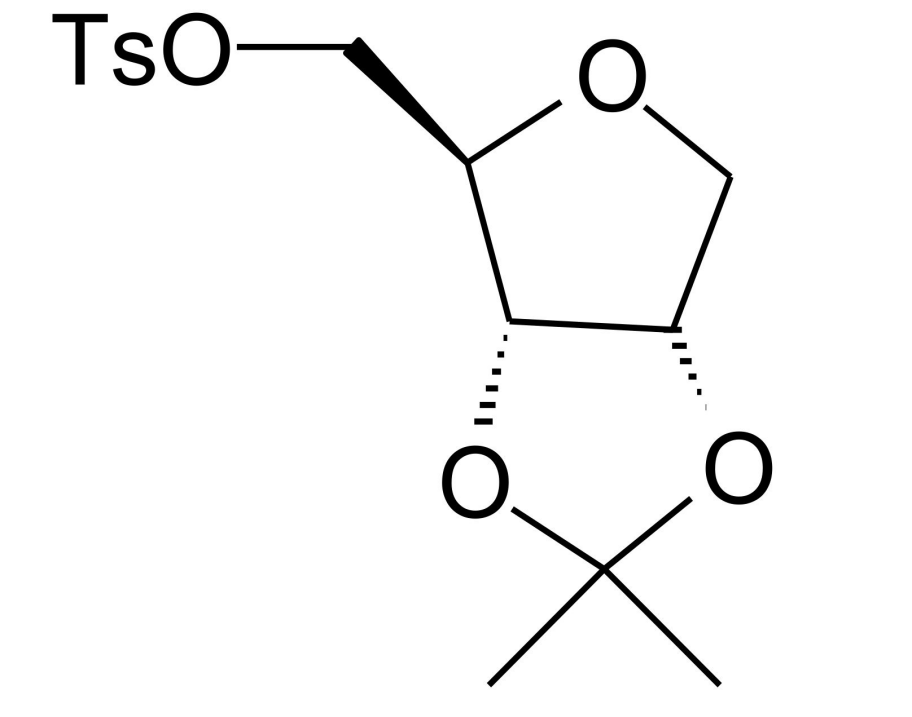	100	40	94	60	95	90	83	45	50
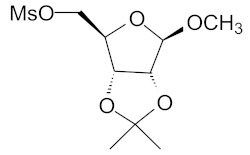	72	19	66	19	55	63	-	-	-
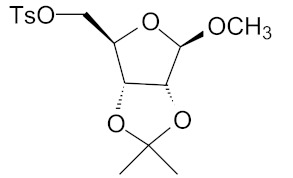	67	28	78	31	70 * 37 **	72	trace	trace	-

Reactions: * at 100 °C solvent-free and ** at 70 °C in acetonitrile.

**Table 2:** Crystal data, data collection and refinement for methyl 2,3-*O*-isopropylidene-5-*O*-mesyl-*β*-D-ribofuranoside (**5**), deposition number CCDC 253950.

Crystal Data	
C_10_H_18_O_7_S	*D*_x_ = 1.399 Mg × m^−3^
*M_r_* = 282.30	Melting point = 345.2–346.2 K
Orthorhombic, *P*2_1_2_1_2_1_	Mo *K*α radiation, λ = 0.71073 Å
*a* = 5.474 (9) Å	Cell parameters from 40 reflections
*b* = 10.840 (5) Å	θ = 3.0–16.4°
*c* = 22.588 (9) Å	μ = 0.26 mm^−1^
*V* = 1340 (2) Å^3^	*T* = 200 K
*Z* = 4	Block, colorless
*F*(000) = 600	0.50 × 0.13 × 0.12 mm
Data collection	
KUMA KM4, point detector diffractometer	θ_max_ = 25.1°, θ_min_ = 1.8°
ω– 2 θ scans	*h* = −2→6
1393 measured reflections	*k* = −5→13
1393 independent reflections	*l* = −11→28
690 reflections with *I* > 2σ (*I*)	3 standard reflections every 200 reflections
*R*_int_ = 0.053	intensity decay: 1.1%
Refinement	
Refinement on *F*^2^	Secondary atom site location: inferred from neighboring sites
Least-squares matrix: full	Hydrogen site location: inferred from neighboring sites
*R*[*F*^2^ > 2σ (*F*^2^)] = 0.069	H-atom parameters constrained
*wR*(*F*^2^) = 0.281	*w* = 1/[σ^2^(*F*_o_^2^) + (0.1511*P*)^2^] where *P* = (*F*_o_^2^ + 2*F*_c_^2^)/3
*S* = 1.08	(Δ/σ)_max_ = 0.001
1393 reflections	Δ>_max_ = 0.44 e Å^−3^
163 parameters	Δ>_min_ = -0.48 e Å^−3^
0 restraints	Absolute structure: No quotients, so Flack parameter determined by classical intensity fit
Primary atom site location: structure-invariant direct methods	Absolute structure parameter: 0.2 (6)

## Supplementary Materials

The following are available online at https://www.mdpi.com/1420-3049/25/9/2161/s1: CCDC 253,950 contains the supplementary crystallographic data for this paper. The data can be obtained free of charge from The Cambridge Crystallographic Data Centre via www.ccdc.cam.ac.uk/structures. 3D Models (Protein Data Bank format) of **4f** and **4g**.

## References

[1] Strecker A. (1868). Ueber eine neue Bildungsweise und die Constitution der Sulfosäuren. Ann. Chem. Pharm..

[2] Tipson R.S. (1953). Sulfonic Esters of Carbohydrates. Adv. Carbohydr. Chem..

[3] Rigby G.W. (1938). Acetone Soluble Cellulose Aryl Sulfonates and Their Preparation. U.S. Patent.

[4] Jeanloz R.W., Jeanloz D.A. (1957). Partial Esterification of Methyl 4,6-O-Benzylidene-α-D-glucopyranoside in Pyridine Solution. J. Am. Chem. Soc..

[5] Feit P.W., Nielsen O.T. (1966). Glycerol 1,3- and 1,2,4-butanetriol 1,4-bismethanesulfonates. J. Med. Chem..

[6] Ball D.H., Parrish F.W. (1968). Sulfonic esters of carbohydrates: Part I. Advances in Carbohydrate Chemistry.

[7] Hochberg M., Chevalier X., Henrotin Y., Hunter D.J., Uebelhart D. (2013). Symptom and structure modification in osteoarthritis with pharmaceutical-grade chondroitin sulfate: What’s the evidence?. Curr. Med. Res. Opin..

[8] McLean J. (1959). The Discovery of Heparin. Circulation.

[9] Petitou M., Duchaussoy P., Lederman I., Choay J., Jacquinet J.C., Sinay P., Torri G. (1987). Synthesis of heparin fragments: A methyl alpha-pentaoside with high affinity for antithrombin III. Carbohydr. Res..

[10] Petitou M., Van Boeckel C.A. (2004). A synthetic antithrombin III binding pentasaccharide is now a drug! What comes next?. Angew. Chem..

[11] Brownlee M. (2001). Biochemistry and molecular cell biology of diabetic complications. Nature.

[12] Spiers A.S.D., Malone H.F. (1966). The significance of serum hexosamine levels in patients with cancer. Br. J. Cancer.

[13] DeBerardinis R.J., Sayed N., Ditsworth D., Thompson C.B. (2008). Brick by brick: Metabolism and tumor cell growth. Curr. Opin. Gen. Dev..

[14] Wike-Hooley J.L., Haveman J., Rheinhold H.S. (1984). The relevance of tumour pH to the treatment of malignant disease. Radiother. Oncol..

[15] Ward K.A., Jain R.K. (1988). Response of tumors to hyperglycemia: Characterization, significance and role in hyperthermia. Int. J. Hyperth..

[16] Tannock I.F., Rotin D. (1989). Acid pH in Tumors and Its Potential for Therapeutic Exploitation. Cancer Res..

[17] Sacoman J.L., Badish L.N., Sharkey T.D., Hollingsworth R.I. (2012). The metabolic and biochemical impact of glucose 6-sulfonate (sulfoquinovose), a dietary sugar, on carbohydrate metabolism. Carbohydr. Res..

[18] Viola R.E. (1984). Kinetic studies of the reactions catalyzed by glucose-6-phosphate dehydrogenase from *Leuconostoc mesenteroides*: pH variation of kinetic parameters. Arch. Biochem. Biophys..

[19] Wong C.H., Gordon J., Cooney C.L., Whitesides G.M. (1981). Regeneration of NAD(P)H Using Glucose-6-Sulfate and Glucose-6-Phosphate Dehydrogenase. J. Org. Chem..

[20] Ray W.J., Long J.W., Owens J.D. (1976). An analysis of the substrate-induced rate effect in the phosphoglucomutase system. Biochemistry.

[21] Rose I.A., Warms J.V.B., Kosow D.P. (1974). Specificity for the glucose-6-*P* inhibition site of hexokinase. Arch. Biochem. Biophys..

[22] Musicki B., Widlanski T.S. (1990). Synthesis of Carbohydrate Sulfonates and Sulfonate Esters. J. Org. Chem..

[23] Kleszczyńska H., Sarapuk J., Różycka-Roszak B. (1998). The Role of Counterions in the Interaction of Some Cationic Surfactants with Model Membranes. Pol. J. Environ. Stud..

[24] Telesiński A., Pawłowska B., Pater J., Biczak R., Śnioszek M. (2017). The role of anion in the impact of tetraethylammonium salts on soil phosphatase activities. Ecol. Quest..

[25] Nowacki A., Dmochowska B., Jączkowska E., Sikora K., Wiśniewski A. (2011). Theoretical studies of the formation of quaternary ammonium mesylates. Comput. Theor. Chem..

[26] Nowacki A., Sikora K., Dmochowska B., Wiśniewski A. (2012). Studies of the formation of *N*-substituted pyridinium mesylates: A theoretical approach. Comput. Theor. Chem..

[27] Sarabia-Garcia F., Lopez-Herrera F.J. (1996). Studies on the synthesis of tunicamycin. The preparation of 7-deoxy-2-deamino-6-hydroxy tunicamine and related products. Tetrahedron.

[28] Dmochowska B., Sikora K., Chojnacki J., Wojnowski W., Wiśniewski A. (2013). *N,N,N*-Trimethyl-*N*-(methyl-5-deoxy-2,3-*O*-isopropylidene-β-D-ribofuranosid-5-yl)ammo-nium 4-methylbenzenesulfonate sesquihydrate. Acta Cryst..

[29] Engineering Toolbox. https://www.engineeringtoolbox.com/.

[30] Skorupa E., Dmochowska B., Pellowska-Januszek L., Wojnowski W., Chojnacki J., Wiśniewski A. (2004). Synthesis and structure of selected quaternary N-(1,4-anhydro-5-deoxy-2,3-O-isopropylidene-D,L-ribitol-5-yl)ammonium salts. Carbohydr. Res..

[31] Stewart J.J.P. (2007). Optimization of parameters for semiempirical methods V: Modification of NDDO approximations and application to 70 elements. J. Mol. Model.

[32] Stewart J.J.P. MOPAC2016. Stewart Computational Chemistry. http://openmopac.net.

